# Combined Quantification and Characterization of Dissolved Organic Matter by Liquid Chromatography–Mass Spectrometry Using Charged Aerosol Detection

**DOI:** 10.1021/jasms.4c00255

**Published:** 2024-10-05

**Authors:** Stacey L. Felgate, Elizabeth Jakobsson, Andrea Balderrama Subieta, Lars J. Tranvik, Jeffrey A. Hawkes

**Affiliations:** 1 Department of Chemistry, Uppsala University, Uppsala 751 23, Sweden; 2 Department of Ecology and Evolution, Uppsala University, Uppsala 752 36, Sweden

**Keywords:** dissolved organic matter, liquid chromatography, mass spectrometry, electrospray ionization, charged aerosol detector, absorbance spectroscopy

## Abstract

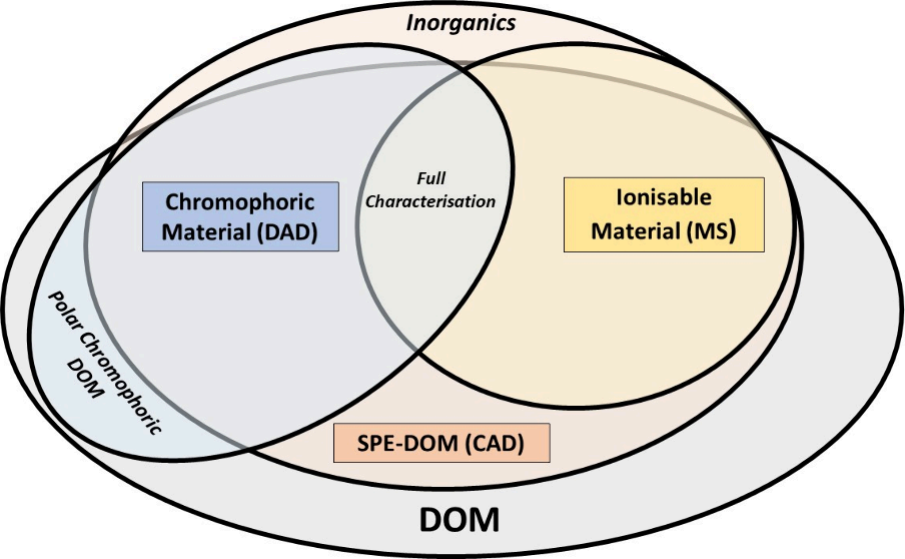

Dissolved organic matter (DOM) is a complex mixture of thousands of molecular formulas comprised of an unknown number of chemical compounds, the concentration and composition of which are critical to ecosystem function and biogeochemical cycling. Despite its importance, our understanding of the DOM composition is lacking. This is principally due to its molecular complexity, which means that no single method is capable of describing DOM in its entirety. Quantification is typically done by proxy (e.g., relative to carbon content) and does not necessarily match well to compositional data, due to incomplete analytical windows and selectivity of different analytical methods. We present an integrated liquid chromatography (LC)–diode array detector (DAD)–charged aerosol detector (CAD)–mass spectrometry (MS) pipeline designed to both characterize and quantify solid-phase extractable DOM (SPE-DOM) in a single analysis. We applied this method to a set of eight Swedish water bodies sampled in the summer and winter. Chromophoric SPE-DOM was proportionally higher in samples with higher SPE-DOM concentrations but remained relatively consistent between sampling occasions. Ionizable SPE-DOM was relatively consistent across sites but was proportionally higher in summer. Overall, the carbon content of DOM was very consistently ∼40% across sites in both summer and winter. These findings suggest that SPE-DOM concentration at these sites is driven by (presumably allochthonous) chromophoric inputs, with an increased relative contribution in summer of material that is more ionizable and less chromophoric and may be either autochthonous or selectively enriched from allochthonous sources. Thus, with minimal additional effort, this method provided further compositional insights not attained by any single analysis in isolation.

## 1 Introduction

Dissolved organic matter (DOM) is an ultracomplex mixture, the movement and processing of which underpin the ecological and biogeochemical functioning of aquatic ecosystems.^1^ Improving our understanding of DOM concentration, chemical composition, and their drivers is of key interest, but the diverse chemistry and molecular complexity of DOM make this difficult.^2,3^

From a compositional perspective, comprehensive analysis is challenging because DOM encompasses a chemical space which is much broader than the analytical windows available to analyze it.^4−6^ One of the most common methods used in DOM characterization is electrospray ionization high resolution mass spectrometry (ESI-HRMS), the analytical window of which is limited to material which is both extractable via solid phase extraction (SPE) and ionizable under electrospray conditions.^6^ Another common window through which DOM is characterized is via absorbance spectrometry over UV–visible wavelengths, which explicitly targets the chromophoric fraction. The relationship among extractable, ionizable, and chromophoric DOM fractions will vary according to the chemical composition of the sample. The extent of their overlap, conceptualized in the TOC graphic, is poorly constrained but likely to be highly variable as the features detected originate from differing functionality,^7^ and the ecological and geochemical sources of these different compound classes can vary geographically and over time.^8^

SPE is typically undertaken using styrene divinylbenzene (Agilent PPL) cartridges^9^ at acidic pH, which preferentially retain hydrophobic compounds while allowing hydrophilic ones, including mono- and polysaccharides, free amino acids and peptides, and basic compounds like amines to escape.^10,11^ Evidence suggests that these hydrophilic analytes are also unsuitable for ESI-MS analysis due to poor ionization potential and/or low mass range,^12,13^ and so despite SPE having variable extraction efficiencies (e.g., 48%;^9^ 70–85%^14^), the material which is lost is unlikely to dramatically alter ESI-HRMS results. In the retained, hydrophobic DOM fraction, the ionization potential largely controls what is observed. For example, high molecular weight chromophoric DOM is well extracted onto PPL but does not ionize well under typical ESI-HRMS conditions.^6^ Thus, compositional data tend to be biased toward more ionizable compound classes such as carboxylic rich alicyclic molecules (CRAM).^6^

A major constraint on data interpretation is that the extent of this ionization bias is unknown, unlike DOM loss as a result of SPE, which can be quantified with relative ease as the difference in carbon content before and after extraction. Extracted DOM that is not measured due to poor ionization efficiency is not easily quantifiable, and standard ESI-HRMS pipelines are not capable of recognizing it and can only determine the extent of ionization suppression by using post column addition of internal standards.^15^

The extent of material ionizability may be better determined by comparing the MS signal with a more quantitative detector such as a diode array detector (DAD) or charged aerosol detector (CAD),^16^ allowing multidetector comparison of material eluting from a chromatography column and assessment of overall material character and abundance. The DAD allows measurement of light absorbance over a range of wavelengths, and this absorbance is linear with concentration according to the Beer–Lambert law (for a given molar absorptivity), and the CAD detector measures the total amount of material in a solution as aerosol particles after drying of solvent and is quantitative with a slightly nonlinear relationship to concentration for nonvolatile compounds.^17^

In this study, we integrated the signal from a DAD for UV absorbance at 254 nm and a CAD for total material abundance into a standard high-performance liquid chromatography (HPLC; sample separation) ESI-HRMS (mass composition of ionizable material) pipeline. We demonstrate how this setup can be used to (a) quantify SPE-DOM and (b) improve SPE-DOM characterization compared with standard ESI-HRMS analyses.

When applied to a set of eight Swedish waterbodies covering a spectrum of DOM concentrations and compositions, this method was able to capture a wide range of complementary information that would have been partially hidden from each analysis, including SPE-DOM concentration, carbon content, and evaluation of overall absorbance and ionization behavior over the elution gradient. Integration of DAD and CAD into standard HPLC-ESI-HRMS analysis therefore has the potential to unlock new quantitative and compositional information with almost no additional experimental effort. The purpose of this study was not to exhaustively interrogate the data produced by this pipeline in this particular sample set but rather to highlight its potential to add value to ESI-HRMS analyses with minimal additional effort.

## 2 Methods

### 2.1 Sample Collection

Duplicate water samples were collected in late summer (September 1– 9, 2020) and late winter (March 16–22, 2021) from eight different sites near Uppsala, Sweden (sites 1–8; Table S1), for which varying DOM concentrations and compositions were expected. Site 1 was a reed pond, sites 2–4 were lake samples, sites 5–7 were river samples, and site 8 was from a wetland. The samples were collected in duplicate and filtered by ∼0.7 μm glass fiber filter (GF/F). Full site descriptions and sampling protocol are provided in Supporting Information.

### 2.2 Sample Preparation

Samples were extracted onto 100 mg Agilent PPL (styrene-divinylbenzene) cartridges following standard methods^9^ in order to desalt and concentrate the samples. To determine [SPE-DOC] and extraction efficiency, 0.25 mL of eluate was pipetted into acid-washed Eppendorf tubes and dried in a SpeedVac vacuum concentrator at room temperature for 3 h. Vials containing the SPE-DOC extracts, along with process blanks, were weighed pre- and postremoval of a 250 μL aliquot. Dry samples were resuspended in 1 mL of Milli-Q water and sonicated for 15 min. For DOC analysis, the 1 mL aliquot was transferred to a 9 mL acid washed precombusted glass vial and made up to 7.5 mL with Milli-Q water. DOC concentrations were measured as nonpurgeable organic carbon using a Shimadzu total carbon analyzer (Shimadzu TOC-L/TNM-L, Kyoto, Japan). [SPE-DOC] was calculated, and extraction efficiency was reported in terms of the original sample concentration as 100 × [SPE-DOC]/[DOC].

Based on [SPE-DOC], sufficient eluate to contain 50 μg of DOC/∼100 μg of DOM was pipetted into a Milli-Q rinsed Eppendorf tube and dried in a Speedvac for 3 h at 30 °C. The dry sample was redissolved in 100 μL of 5% acetonitrile (acetonitrile) in Milli-Q water + 0.1% formic acid and brought back into solution by vortexing then sonicating for 10 min. This produced a sample with a target concentration of 1000 mg L^–1^ DOM, assuming a 50% carbon content. The supernatant (∼80 μL) was pipetted into a precombusted autosampler vial (1.5 mL with a 300 μL insert). Further details and a full list of reagents used are provided in Supporting Information.

### 2.3 Sample Analysis

**Figure 1:**
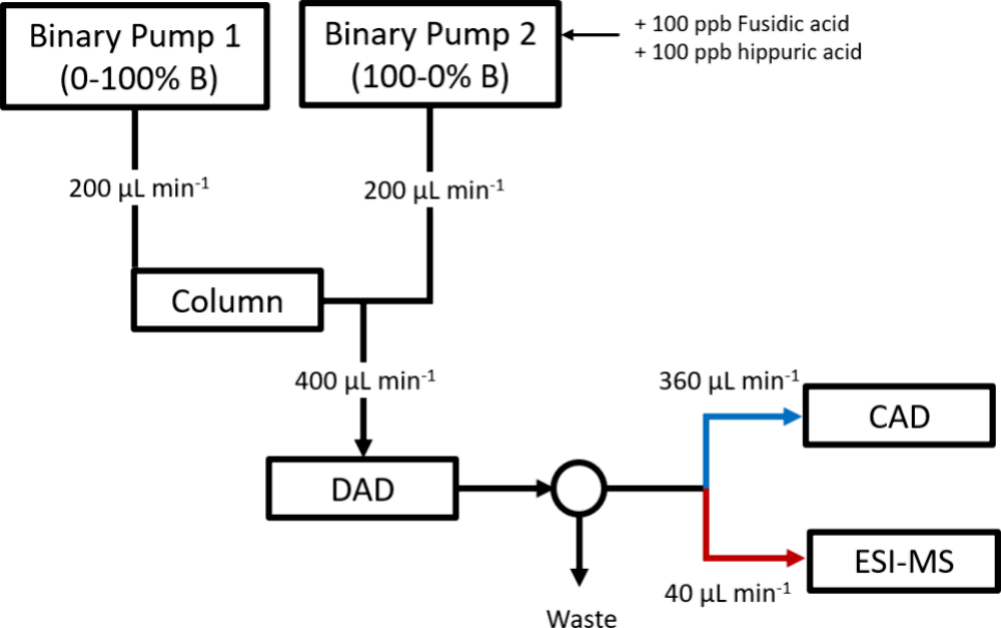
Schematic diagram illustrating setup of the dual-pump HPLC-DAD-CAD-ESI-HRMS method.

DOC and SPE-DOC were both quantified on a Shimadzu TOC-V analyzer, the details of which are provided in Supporting Information. HPLC-DAD-CAD-ESI-HRMS analysis was carried out using an Agilent 1100 series HPLC with an integrated diode array detector (DAD), which was set to measure wavelength 254 nm and which was subsequently connected to a Dionex Corona Ultra RS charged aerosol detector (CAD; for eluting material abundance) and an Orbitrap Velos Pro mass spectrometer (Thermo Fisher, Bremen, Germany) with an electrospray ionization (ESI) source (Figure 1). 254 nm was chosen as the wavelength due to its correlation with DOC concentration and its common usage for quantification of DOM and aromaticity.^18^

We injected 30 μL of each sample, assuming that each had an SPE-DOC concentration of ∼500 mg L^–1^ C and therefore expecting an injection of 15 μg carbon on the column. We determined that it was important to inject similar sample amounts of DOM between samples after a calibration series of SRNOM over 1–40 μg of material was injected, which led to very different peak assignment numbers and average metrics (Table S2). Carryover was very minor, as shown by analysis of a blank after a sample (Figure S1).

Analytes were separated by reversed phase chromatography using a Kinetex Polar C18 column (100 mm × 2.1 mm, 2.6 μm, Phenomenex, Torrance, USA) and a flow rate of 200 μL min^–1^. The separation was conducted over a 15 min gradient from 0 to 100% B (Table S3) using two mobile phases (A = 0.1% formic acid in LCMS grade H_2_O (i.e., 100 mL H_2_O + 100 μL formic acid); B = 0.1% formic acid in ACS grade acetonitrile). A second pump provided a counter gradient postcolumn,^19,20^ which balanced the solvent composition to a constant 50% acetonitrile. 90% of the total flow was diverted to the CAD, running at a pressure of 35 psi with “range” set to 50 pA. The remaining 10% flow was diverted to the ESI source for analysis by HRMS. The ESI was set to −3 kV at 100 °C, and the MS was set to collect data at a resolution of 60 000 (not the maximum of 100 000) in order to collect more transients. The MS was optimized in negative ion mode using direct infusion of a 20 ppm of SRNOM solution in 50% methanol, tuning ion optics to maximize the intensity of the ions at ∼369.1. Hippuric and fusidic acids were added as spike standards to mobile phases attached to the second pump at a final concentration of 100 ppb for each. These exact masses are typically absent in DOM samples, allowing continuous “lock mass” calibration at two *m*/*z* values (178.050 97 and 515.337 81), much improved mass accuracy, and would be available for normalization of the MS signal to account for ionization suppression,^15^ although this was not done in this study.

### 2.4 Data Processing and Analysis

#### 2.4.1 CAD Calibration

CAD peak area data (the area underneath the CAD signal) were calibrated against [DOM] using a 1000 mg L^–1^ SRNOM solution prepared with 5% acetonitrile in pure (Milli-Q) water. This was injected at 1, 5, 10, 20, 30, 40, 50, and 60 μL volumes (equivalent to 1–60 μg of DOM). CAD peak areas for each injection volume were integrated (baseline corrected) between 1.5 and 11.5 min. The resultant quadratic calibration curve had an *R*^2^ of 0.996 (Figure S2; eq 1), allowing injected [DOM] to be predicted from the CAD peak area according to its inverse equation (eq 2):
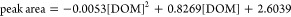
1
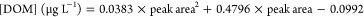
2

#### 2.4.2 MS Data

MS data were converted to mzXML files for processing in MATLAB. A formula assignment routine was written in MATLAB (R2023a, Mathworks), which is available along with raw sample data in Supporting Information. Formulas were assigned when within 2 ppm of a theoretical formula mass between 120 and 800 Da, with the following constraints: C = 4–40, H = 4–80, O = 0–35, N = 0–1, S = 0–1, ^13^C = 0–1, H/C = 0.3–2.4, and O/C = 0–1, and double bond equivalence-oxygen > −10. Formulas were not allowed to contain more than one of N, S, and ^13^C. Noise was removed using the Kendrick mass defect slice method,^21^ and high molecular weight doubly charged interference peaks were removed from consideration before assignment.^22^ In total, eight blanks were measured, and these were used for MS data processing, as the assigned formula intensities were required to be more than 3× as high as the average blank to be considered as a sample peak. A summary of assignment errors can be found for an example sample in Figure S3, and the appended MATLAB code can be used to find and plot errors for the other samples. An assessment of assignment coverage is also shown for three samples in Figure S4.

## 3 Results and Discussion

### 3.1 Carbon Concentration Analyses and Charged Aerosol Detection

**Figure 2:**
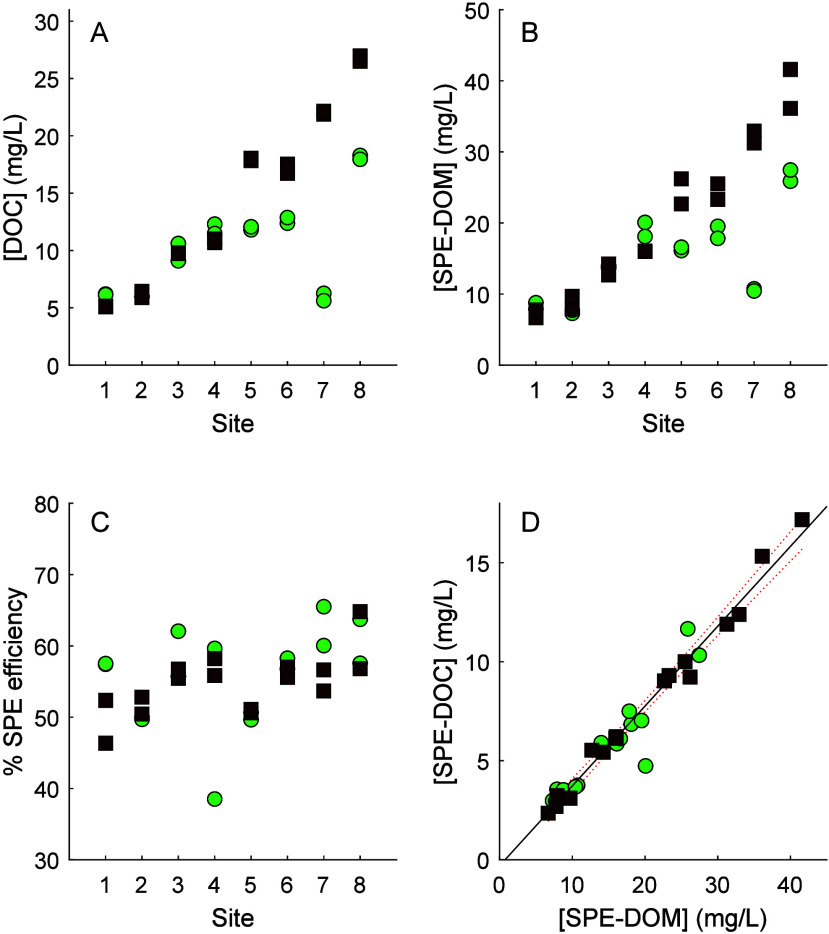
(A) DOC concentration (measured by Shimadzu TOC analyzer); (B) SPE-DOM concentrations (measured by CAD); (C) SPE efficiency (%; measured by Shimadzu TOC analyzer before and after extraction; (D) SPE-DOM vs SPE-DOC, equation, SPE-DOC = 0.403[SPE-DOM] – 0.30, *r*^2^ = 0.96. Each site is shown for summer 2020 (green circles) and winter 2021 (brown squares). For each sampling occasion, each of the two replicates are shown.

DOC concentration ([DOC]; Figure 2A) ranged from 5.11 to 26.74 mg L^–1^ (mean ± SD = 12.39 ± 6.40 mg L^–1^). [SPE-DOM] (Figure 2B) ranged from 6.68 to 41.60 mg L^–1^ (17.90 ± 9.21 mg L^–1^). Both varied significantly at the 95% confidence interval across the chosen sites by season (Wilcoxon signed rank test; *p* = 0.0494; *p* = 0.0386), and both were clearly higher at sites 5–8 (river and wetland sites) in winter compared to summer. Mean SPE efficiency (Figure 2C) was 55 ± 5% (range = 37–62%). This did not vary with season (Wilcoxon signed rank test; *p* = 0.255) and was consistent between replicates (mean = 2.7 ± 2.6%) with the exception of sample S4 which had a 21.1% difference. This is likely due to some form of contamination or inconsistency in sample preparation and emphasizes the need to process replicate samples (ideally *n* > 2), a practice which is rare in MS studies of DOM.

The comparison in response between [SPE-DOC] by TOC analysis and [SPE-DOM] by CAD analysis in the single HPLC based analytical approach was very favorable; a linear relationship was found with *r*^2^ = 0.96, indicating that 40.3% of SPE-DOM was carbon across sites and seasons. The formal TOC analysis is undertaken on an aqueous sample in a vial and uses a wet combustion method on a platinum catalyst, with detection of oxidized organic carbon as CO_2_ using IR absorption, while the CAD method used here detects matter eluting from the column separation, in solid state using an electrometer after drying of solvent. In other words, the methods are totally independent of each other, and so the high linearity is remarkable and indicates that the HPLC-CAD method can be used in place of SPE-DOC analysis using TOC analyzer, which is time-consuming and requires several handling steps that can lead to contamination. Quantification of SPE-DOM also allowed us to determine % C without the need for an elemental analyzer. The result that ∼40% [SPE-DOM] was carbon was in perfect agreement with a recent study which found that coastal SPE-DOM extracted onto C_18_ contained ∼40% carbon,^23^ assuming uniform CAD response for all DOM.

### 3.2 ESI-MS Analysis

SPE-DOM eluted over a ∼9 min window in the 15 min reversed phase gradient method, and formulas were assigned to each acquired transient in an in-house MATLAB code. In this case, the assigned formula intensities were summed across the chromatogram to provide a single intensity list, similar to what would be acquired by direct infusion but with better coverage of more polar, higher oxygen-containing compounds.^19,24^ Note that it is trivial to fractionate the data *in silico* to various polarity fractions,^19,20,24^ which can be quantified by integrating CAD data. Note that chemical interpretation of such fractions may be problematic due to the complexity of the mixture and the coelution of so many components. It is also possible to analyze the data using software such as MZmine,^25,26^ allowing various types of more in-depth evaluations of the eluting features.

**Figure 3:**
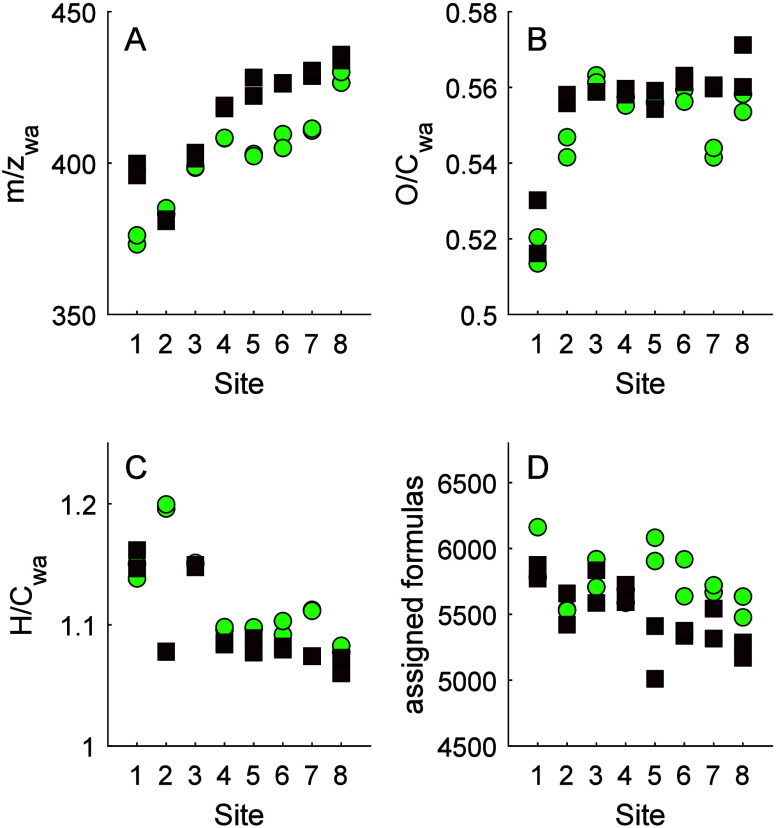
(A) weighted average *m*/*z*; (B) weighted average O/C; (C) weighted average of the o/c; (D) assigned formulas. Each site is shown for summer 2020 (filled circles) and winter 2021 (unfilled squares). Each site is shown for summer 2020 (green circles) and winter 2021 (brown squares). For each sampling occasion, each of two replicates are shown.

Generally, the weighted average mass (*m*/*z*_wa_) and O/C_wa_ of material increased with DOC (i.e., from high water retention time lakes through to the wetland pond (Figure 3A,B), and hydrogen saturation decreased. There was also a clear difference between summer and winter; SPE-DOM had a higher *m*/*z* ratio in winter than summer (414.52 ± 17.54 vs 401.88 ± 15.53; Wilcoxon signed rank test *p* < 0.001), with a higher O/C ratio (0.555 ± 0.013 vs 0.549 ± 0.014; Wilcoxon signed rank test *p* = 0.0061) and a lower H/C ratio (1.096 ± 0.033 vs 1.122 ± 0.037; Wilcoxon signed rank test *p* = 0.0045). We detected, on average, 4.4% fewer peaks in winter than in summer (Figure 3D), and this difference was significant (Wilcoxon signed rank test *p* < 0.01). Taken together, these metrics indicate that in the summer, the ionizable SPE-DOM pool was imprinted with a slight increase in compound diversity, with lighter, less oxygenated, and more saturated material. This was particularly the case at the low water residence time sites with high DOC and was less apparent at the low DOC lakes and ponds.

### 3.3 CAD, DAD, and Total Ion Current

The data collected with the presented method can be interrogated based on elution profiles. Having three simultaneous detectors allows comparison of various chemical properties; in this case, material abundance, absorbance (at chosen wavelengths; here 254 nm), and ESI-MS total assigned current (TAC). As discussed later, the ratios of the various signals can also be investigated.

Integrated CAD signal (1.5–11.5 min), used in this study to determine [SPE-DOM], averaged at 2.87 ± 0.35 (mean ± SD), confirming that efforts to inject a consistent amount of SPE-DOM were largely successful, albeit with a small range of 2.33–3.86. This range represents the uncertainty introduced during sample preparation, which we attribute to the inherent difficulty of working with small volumes (tens of μL) of highly concentrated sample, along with errors in original [DOC] concentration measurement and possibly variance in volatility of DOM, which is the main inaccuracy with CAD measurement.^27^ Here we repeat our recommendation to include sufficient replicates (*n* > 2) in future studies to allow uncertainties to be adequately quantified when reporting these data.

As reported in previous HPLC studies,^19,24,28^ each *m*/*z* peak in DOM typically elutes according to a smooth and gradual elution, deviations from which represent higher abundance of individual isomers. These chromatographically resolved compounds elute sharply at a given time point, and visualization of detector data or detector ratio data over the elution window can in principle help to identify such resolved compounds. Certainly, they can be determined from extracted ion chromatograms (XICs) in software such as MZmine or XCMS if they are ionizable, making these data compatible with metabolomics pipelines. Detection of well resolved chromophoric compounds that do not ionize is also possible with the multidetector approach we promote and may warrant fractionation and isolation of compounds for characterization by other methods. Typically, ESI-MS and DAD detectors are far more sensitive than quantitative detectors like CAD, so single isomers that can be resolved chromatographically can probably not be quantified within DOM (without standards) by using current CAD technology.

**Figure 4:**
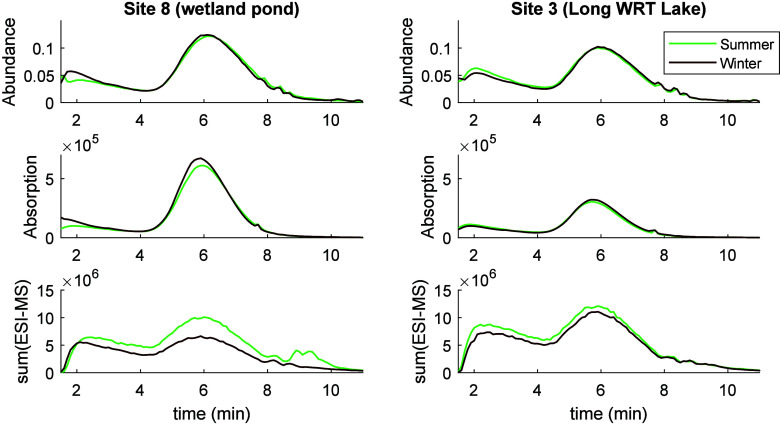
Example data from site 8 (wetland pond), left, and site 3 (a long water retention time lake). The three detector signals are shown, top to bottom: abundance (from CAD), absorption (from DAD), and total assigned current (sum of ESI-MS). Data are plotted between 1.5 and 11 min and are shown for one replicate of each season; see legend.

At both sites shown in Figure 4, abundance distributions by polarity, as shown by CAD, were very similar between seasons and were similar in scale between the two sites, all indicating that similar amounts of DOM were injected, as intended. At site 8, a wetland pond with very high DOC, UV absorbance was somewhat higher than at site 3, a long water retention time lake. This is in line with expectations; i.e., specific UV absorbance per DOC concentration should be lower at a longer water retention time site like site 3. In both cases, the abundance of UV absorbing material was very slightly higher in winter but with similar polarity profiles, indicating similar composition but differing abundance. Specifically, for site 8 the apex position of UV absorbance was at 6.1 × 10^5^, 5.9 min in summer and 6.7 × 10^5^, 5.9 min in winter. At site 3, the apex was at 3.0 × 10^5^, 5.7 min in summer and 3.2 × 10^5^, 5.7 min in winter (Figure 4).

The distribution of the ionizable material varied more between summer (higher) and winter (lower). The increase in ionizable material in summer offset the relative decrease in chromophoric material, resulting in a similar overall abundance of DOM injected. This suggests that ionizable and chromophoric DOM are different pools of matter, at least in the seasonally variable DOM. There was more ionizable material at site 3 than site 8, and the retention behavior suggests that site 3 had slightly more hydrophilic SPE-DOM (Figure 4).

**Figure 5:**
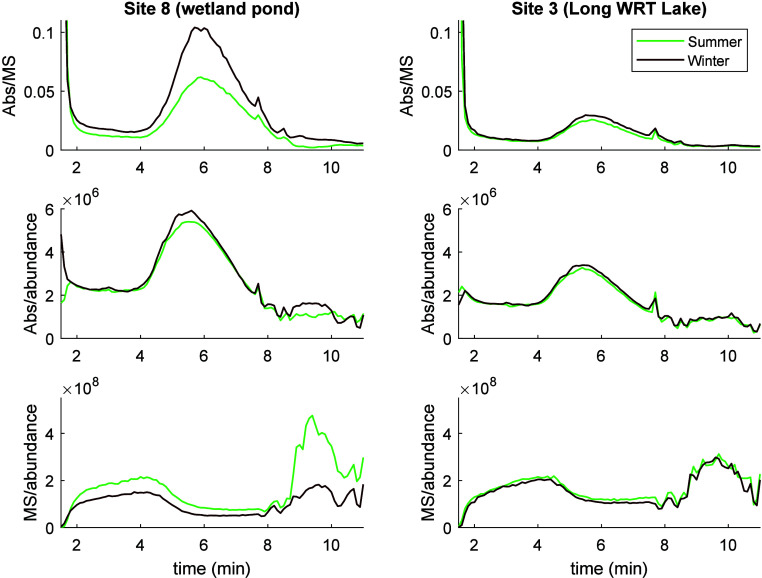
Chromatograms showing the ratio of absorbance to ionizable material (top), absorbance to abundance (middle), and ionizable material to abundance (bottom) for the two sites shown in Figure 4. Summer and winter are shown in green and black; see legend. Note that CAD data have been normalized to zero (CAD_t_ – CAD_t11 min_) to remove negative numbers for the purpose of calculating ratios.

Observations of differences between detectors can be examined more closely by looking at the ratios of these parameters (Figure 5). The absorbance/ionization ratio (Abs/MS) was considerably higher in winter than in summer for site 8, which signifies that SPE-DOM at this site was more chromophoric but less ionizable in winter than in summer, particularly at midpolarity ranges from 5 to 8 min retention. Interestingly, the absorbance to abundance ratio in the same polarity region was only marginally higher in winter, while the MS signal to abundance ratio was somewhat lower. This suggests that the absorbance to ionizability ratio was largely controlled by the loss of ionizable material during the winter rather than increase per μg of DOM of chromophoric material. It suggests that ionizable material is more seasonally dynamic, in terms of composition, but that overall DOC concentration (and carbon flux) is largely dictated by chromophoric DOM. These effects are particularly pronounced at the high DOC sites, like site 8, and are rather minor at high water retention sites, like site 3, where DOM dynamics are perhaps less seasonally pronounced, being averaged over longer time scales.

**Figure 6:**
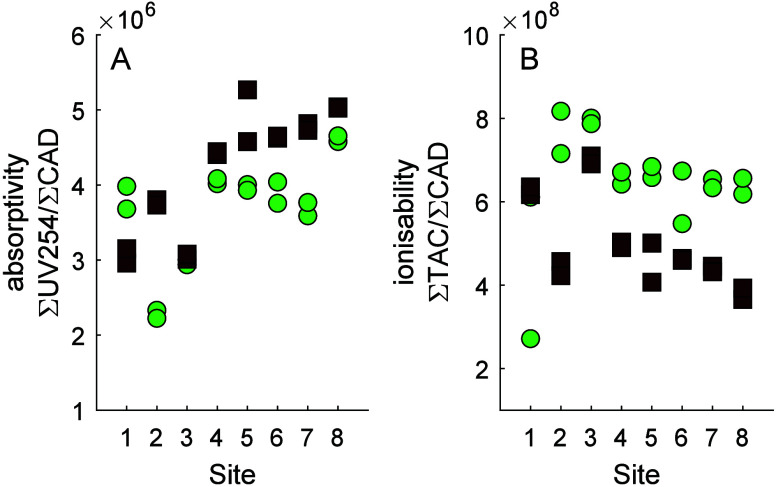
(A) absorptivity (sum of UV absorbance divided by CAD signal); (B) ionizability (sum of total assigned current in MS divided by CAD signal). Each site is shown for summer 2020 (green circles) and winter 2021 (brown squares). For each sampling occasion, each of two replicates are shown.

Where standard ESI-HRMS findings combine to suggest that SPE-DOM from eight Swedish waterbodies was heavier, more highly unsaturated, and less diverse in winter than in summer, the inclusion of a CAD and DAD added layers of additional context. At some sites, we found that SPE-DOM was proportionally more chromophoric in winter than in summer and more ionizable in summer than in winter (Figure 6). The apparent increased ionizability was likely due to alleviation of ionization suppression because the internal standards (hippuric and fusidic acid) were also found at higher intensity in the summer samples compared with winter (Figure S5). The fact that SPE-DOM was >50% more ionizable in summer than in winter at some sites is an important finding in relation to other studies in which samples are obtained at different times of year and in which the degree of ionization or the extent of ionization suppression is not typically considered,^15,20^ much less quantified.

### 3.4 Future Prospects for Combined Quantity and Quality Data

High resolution techniques can give very rich data that provide important novel information about character and chemical structure of DOM,^29^ which can help to determine source and reactivity.^30^ However, geographical models that aim to utilize information about source and reactivity of DOM also require information about abundance in order to determine flux.^31^ For this reason, features such as ionization efficiency and knowledge about whether or not chromophoric DOM is also ionizable must be taken into consideration. For this reason, methods that allow separation of organic matter into different components and also provide information about both the character and the abundance of these components are incredibly useful.

Geographical models of DOM abundance are limited to very few components/pools of organic matter, due to current computational limitations,^31^ and it is difficult to reduce complex DOM data into few enough components. Typically, models use only 2–3 DOM pools, with varying absorption characteristics and proposed sources, in order to explain DOC concentration changes over time and space.^31,32^ Methods using chromatographic separation of DOM are promising in this regard because the inclusion of a chemical characteristic (polarity with C_18_, size with size exclusion, etc.) allows definition of chemical components in a multiway statistical approach, similar to PARAFAC.^33^ Components can overlap, if necessary, and can contain rich information about the chemical formula ion abundance or light absorbance. Both reversed phase and size exclusion separations have made promising advances in this regard, and the presented method adds the possibility of quantification to our previous method using reversed phase separation^33^ or adds mass spectrometry to previous possibilities using LC-OCD.^34−36^ In future work, we expect to be able to include reactivity experiments (e.g., to UV light and biological decay) in order to determine the relative loss of material (CAD), chromophores (DAD), and ionizable material (ESI-MS) from SPE-DOM under different biogeochemical conditions,^35^ allowing testing of previously proposed stability of model DOM fractions.^31^

## 4 Conclusions

In the presented study, we have demonstrated a new method with which to quantify SPE-DOM during LC-MS analysis using data obtained from an inline charged aerosol detector (CAD). This method also provides the ability to quantify the carbon content of a SPE-DOM sample without any additional elemental analysis. Furthermore, we have shown that new levels of insight can be gained by including a CAD and diode array detector (DAD) into the standard HPLC-ESI-HRMS DOM analysis pipeline. We demonstrate this by highlighting how the presence and/or absence in CAD, DAD, and peaks in the total ion current or total assigned current can be interpreted to identify differences in how chromophoric and/or ionizable extractable DOM is.

This analysis could be further improved by adding tandem MS/MS to target analytes eluting as chromatographic peaks in the mass spectral data, allowing the compound(s) responsible for sharply eluting peaks in XICs to be matched to spectral libraries. There is exciting potential for this type of data set to be used to validate and improve our understanding of DOM dynamics in the environment.

## Data Availability

All data used in this study are included in the manuscript and Supporting Information.

## Supporting Information Available

All data processing code, raw MS, UV, and CAD data files, and supplementary materials (Text S1–S5, Tables S1–S6, and Figures S1–S8) (PDF)

## References

[ref1] Raymond P. A.; Spencer R. G. M. Riverine DOM. In Biogeochemistry of Marine Dissolved Organic Matter, 2nd ed.; Elsevier, 2015; 10.1016/B978-0-12-405940-5.00011-X.

[ref2] Kothawala D. N.; Kellerman A. M.; Catalán N.; Tranvik L. J. Organic Matter Degradation across Ecosystem Boundaries: The Need for a Unified Conceptualization. Trends Ecol. Evol. 2021, 36, 113 10.1016/j.tree.2020.10.006. 33168153

[ref3] Xenopoulos M. A.; Barnes R. T.; Boodoo K. S.; Butman D.; Catalán N.; D’Amario S. C.; Fasching C.; Kothawala D. N.; Pisani O.; Solomon C. T.; Spencer R. G. M.; Williams C. J.; Wilson H. F. How Humans Alter Dissolved Organic Matter Composition in Freshwater: Relevance for the Earth’s Biogeochemistry. Biogeochemistry 2021, 154, 323 10.1007/s10533-021-00753-3.

[ref4] Kew W.; Mackay C. L.; Goodall I.; Clarke D. J.; Uhrín D. Complementary Ionization Techniques for the Analysis of Scotch Whisky by High Resolution Mass Spectrometry. Anal. Chem. 2018, 90 (19), 11265–11272. 10.1021/acs.analchem.8b01446. 30188688

[ref5] Wünsch U. J.; Geuer J. K.; Lechtenfeld O. J.; Koch B. P.; Murphy K. R.; Stedmon C. A. Quantifying the Impact of Solid-Phase Extraction on Chromophoric Dissolved Organic Matter Composition. Marine Chemistry 2018, 207, 33–41. 10.1016/j.marchem.2018.08.010.

[ref6] Patriarca C.; Balderrama A.; Može M.; Sjöberg P. J. R.; Bergquist J.; Tranvik L. J.; Hawkes J. A. Investigating the Ionization of Dissolved Organic Matter by Electrospray. Anal. Chem. 2020, 92 (20), 14210–14218. 10.1021/acs.analchem.0c03438. 32940031 PMC7584329

[ref7] Hertkorn N.; Harir M.; Cawley K. M.; Schmitt-Kopplin P.; Jaffé R. Molecular Characterization of Dissolved Organic Matter from Subtropical Wetlands: A Comparative Study through the Analysis of Optical Properties, NMR and FTICR/MS. Biogeosciences 2016, 13 (8), 2257–2277. 10.5194/bg-13-2257-2016.

[ref8] Spencer R. G. M.; Butler K. D.; Aiken G. R. Dissolved Organic Carbon and Chromophoric Dissolved Organic Matter Properties of Rivers in the USA. J. Geophys. Res.: Biogeosci. 2012, 117 ( (G3), ), 10.1029/2011JG001928.

[ref9] Dittmar T.; Koch B.; Hertkorn N.; Kattner G. A Simple and Efficient Method for the Solid-Phase Extraction of Dissolved Organic Matter (SPE-DOM) from Seawater. Limnol. Oceanogr.: Methods 2008, 6 (6), 230–235. 10.4319/lom.2008.6.230.

[ref10] Raeke J.; Lechtenfeld O. J.; Wagner M.; Herzsprung P.; Reemtsma T. Selectivity of Solid Phase Extraction of Freshwater Dissolved Organic Matter and Its Effect on Ultrahigh Resolution Mass Spectra. Environ. Sci.: Processes Impacts 2016, 18 (7), 918–927. 10.1039/C6EM00200E. 27363664

[ref11] Hawkes J. A.; Rossel P. E.; Stubbins A.; Butterfield D.; Connelly D. P.; Achterberg E. P.; Koschinsky A.; Chavagnac V.; Hansen C. T.; Bach W.; Dittmar T. Efficient Removal of Recalcitrant Deep-Ocean Dissolved Organic Matter during Hydrothermal Circulation. Nat. Geosci. 2015, 8 (11), 856–860. 10.1038/ngeo2543.

[ref12] Lechtenfeld O. J.; Kattner G.; Flerus R.; McCallister S. L.; Schmitt-Kopplin P.; Koch B. P. Molecular Transformation and Degradation of Refractory Dissolved Organic Matter in the Atlantic and Southern Ocean. Geochim. Cosmochim. Acta 2014, 126, 321–337. 10.1016/j.gca.2013.11.009.

[ref13] Grasset C.; Groeneveld M.; Tranvik L. J.; Robertson L. P.; Hawkes J. A. Hydrophilic Species Are the Most Biodegradable Components of Freshwater Dissolved Organic Matter. Environ. Sci. Technol. 2023, 57 (36), 13463–13472. 10.1021/acs.est.3c02175. 37646447 PMC10501193

[ref14] Volikov A. B.; Sobolev N. A.; Khreptugova A. N.; Perminova I. V. Static and Dynamic Sorption of DOM on Bond Elute PPL and Bondesil PPL Sorbents: Physical-Chemical Characteristics. Sep. Sci. Technol. 2023, 58 (4), 642–653. 10.1080/01496395.2022.2145224.

[ref15] Rodrigues Matos R.; Jennings E. K.; Kaesler J.; Reemtsma T.; Koch B. P.; Lechtenfeld O. J. Post Column Infusion of an Internal Standard into LC-FT-ICR MS Enables Semi-Quantitative Comparison of Dissolved Organic Matter in Original Samples. Analyst 2024, 149 (12), 3468–3478. 10.1039/D4AN00119B. 38742449

[ref16] Cole R.; Freeto S.; Gamache P.; Woodcock M.; McCarthy R.; Laws K.; Asa D. HPLC Analysis of Nonvolatile Analytes Using Charged Aerosol Detection. LCGC North America 2005, 23 (2), 150–161.

[ref17] Górecki T.; Lynen F.; Szucs R.; Sandra P. Universal Response in Liquid Chromatography Using Charged Aerosol Detection. Anal. Chem. 2006, 78 (9), 3186–3192. 10.1021/ac060078j. 16643012

[ref18] Weishaar J. L.; Aiken G. R.; Bergamaschi B. A.; Fram M. S.; Fujii R.; Mopper K. Evaluation of Specific Ultraviolet Absorbance as an Indicator of the Chemical Composition and Reactivity of Dissolved Organic Carbon. Environ. Sci. Technol. 2003, 37 (20), 4702–4708. 10.1021/es030360x. 14594381

[ref19] Han L.; Kaesler J.; Peng C.; Reemtsma T.; Lechtenfeld O. J. Online Counter Gradient LC-FT-ICR-MS Enables Detection of Highly Polar Natural Organic Matter Fractions. Anal. Chem. 2021, 93 (3), 1740–1748. 10.1021/acs.analchem.0c04426. 33370097

[ref20] Lechtenfeld O. J.; Kaesler J.; Jennings E. K.; Koch B. P. Direct Analysis of Marine Dissolved Organic Matter Using LC-FT-ICR MS. Environ. Sci. Technol. 2024, 58 (10), 4637–4647. 10.1021/acs.est.3c07219. 38427796 PMC10938638

[ref21] Schum S. K.; Brown L. E.; Mazzoleni L. R. MFAssignR: Molecular Formula Assignment Software for Ultrahigh Resolution Mass Spectrometry Analysis of Environmental Complex Mixtures. Environ. Res. 2020, 191, 110114 10.1016/j.envres.2020.110114. 32866496

[ref22] Patriarca C.; Hawkes J. A. High Molecular Weight Spectral Interferences in Mass Spectra of Dissolved Organic Matter. J. Am. Soc. Mass Spectrom. 2021, 32 (1), 394–397. 10.1021/jasms.0c00353. 33232162 PMC7791554

[ref23] Felgate S. L.; Craig A. J.; Moodie L. W. K.; Hawkes J. Characterization of a Newly Available Coastal Marine Dissolved Organic Matter Reference Material (TRM-0522). Anal. Chem. 2023, 95 (16), 6559–6567. 10.1021/acs.analchem.2c05304. 37052954 PMC10134136

[ref24] Patriarca C.; Bergquist J.; Sjöberg P. J. R.; Tranvik L.; Hawkes J. A. Online HPLC-ESI-HRMS Method for the Analysis and Comparison of Different Dissolved Organic Matter Samples. Environ. Sci. Technol. 2018, 52 (4), 2091–2099. 10.1021/acs.est.7b04508. 29241333

[ref25] Pluskal T.; Castillo S.; Villar-briones A.; Oresic M. MZmine 2 : Modular Framework for Processing, Visualizing, and Analyzing Mass Spectrometry- Based Molecular Profile Data. BMC Bioinf. 2010, 11, 395 10.1186/1471-2105-11-395. PMC2918584 20650010

[ref26] Schmid R.; Heuckeroth S.; Korf A.; Smirnov A.; Myers O.; Dyrlund T. S.; Bushuiev R.; Murray K. J.; Hoffmann N.; Lu M.; Sarvepalli A.; Zhang Z.; Fleischauer M.; Dührkop K.; Wesner M.; Hoogstra S. J.; Rudt E.; Mokshyna O.; Brungs C.; Ponomarov K.; Mutabdžija L.; Damiani T.; Pudney C. J.; Earll M.; Helmer P. O.; Fallon T. R.; Schulze T.; Rivas-Ubach A.; Bilbao A.; Richter H.; Nothias L.-F.; Wang M.; Orešič M.; Weng J.-K.; Böcker S.; Jeibmann A.; Hayen H.; Karst U.; Dorrestein P. C.; Petras D.; Du X.; Pluskal T. Integrative Analysis of Multimodal Mass Spectrometry Data in MZmine 3. Nat. Biotechnol. 2023, 41 (4), 447–449. 10.1038/s41587-023-01690-2. 36859716 PMC10496610

[ref27] Ligor M.; Studzińska S.; Horna A.; Buszewski B. Corona-Charged Aerosol Detection: An Analytical Approach. Crit. Rev. Anal. Chem. 2013, 43 (2), 64–78. 10.1080/10408347.2012.746134.

[ref28] Namjesnik-Dejanovic K.; Cabaniss S. E. Reverse-Phase HPLC Method for Measuring Polarity Distributions of Natural Organic Matter. Environ. Sci. Technol. 2004, 38 (4), 1108–1114. 10.1021/es0344157. 14998025

[ref29] Hertkorn N.; Ruecker C.; Meringer M.; Gugisch R.; et al. High-Precision Frequency Measurements : Indispensable Tools at the Core of the Molecular-Level Analysis of Complex Systems. Anal. Bioanal. Chem. 2007, 389, 1311–1327. 10.1007/s00216-007-1577-4. 17924102 PMC2259236

[ref30] D’Andrilli J.; Cooper W. T.; Foreman C. M.; Marshall A. G. An Ultrahigh-Resolution Mass Spectrometry Index to Estimate Natural Organic Matter Lability. Rapid Commun. Mass Spectrom. 2015, 29 (24), 2385–2401. 10.1002/rcm.7400. 26563709 PMC4654268

[ref31] Anderson T. R.; Rowe E. C.; Polimene L.; Tipping E.; Evans C. D.; Barry C. D. G.; Hansell D. A.; Kaiser K.; Kitidis V.; Lapworth D. J.; Mayor D. J.; Monteith D. T.; Pickard A. E.; Sanders R. J.; Spears B. M.; Torres R.; Tye A. M.; Wade A. J.; Waska H. Unified Concepts for Understanding and Modelling Turnover of Dissolved Organic Matter from Freshwaters to the Ocean: The UniDOM Model. Biogeochemistry 2019, 146 (2), 105–123. 10.1007/s10533-019-00621-1.

[ref32] Carter H. T.; Tipping E.; Koprivnjak J.-F.; Miller M. P.; Cookson B.; Hamilton-Taylor J. Freshwater DOM Quantity and Quality from a Two-Component Model of UV Absorbance. Water Res. 2012, 46 (14), 4532–4542. 10.1016/j.watres.2012.05.021. 22698253

[ref33] Wünsch U. J.; Hawkes J. A. Mathematical Chromatography Deciphers the Molecular Fingerprints of Dissolved Organic Matter. Analyst 2020, 145 (5), 1789–1800. 10.1039/C9AN02176K. 31950125

[ref34] Huber S. A.; Balz A.; Abert M.; Pronk W. Characterisation of Aquatic Humic and Non-Humic Matter with Size-Exclusion Chromatography - Organic Carbon Detection - Organic Nitrogen Detection (LC-OCD-OND). Water Res. 2011, 45 (2), 879–885. 10.1016/j.watres.2010.09.023. 20937513

[ref35] Vignola M.; Lenselink J.; Quinn D.; Ijaz U. Z.; Pereira R.; Sloan W. T.; Connelly S.; Moore G.; Gauchotte-Lindsay C.; Smith C. J. Differential Utilisation of Dissolved Organic Matter Compound Fractions by Different Biofilter Microbial Communities. AQUA—Water Infrastruct., Ecosyst. Soc. 2023, 72 (10), 1837–1851. 10.2166/aqua.2023.036.

[ref36] Pereira R.; Isabella Bovolo C.; Spencer R. G. M.; Hernes P. J.; Tipping E.; Vieth Hillebrand A.; Pedentchouk N.; Chappell N. A.; Parkin G.; Wagner T. Mobilization of Optically Invisible Dissolved Organic Matter in Response to Rainstorm Events in a Tropical Forest Headwater River. Geophys. Res. Lett. 2014, 41 (4), 1202–1208. 10.1002/2013GL058658.

