# Supplementary material for: Combined Quantification and Characterization of Dissolved Organic Matter by Liquid Chromatography–Mass Spectrometry Using Charged Aerosol Detection
Source: J Am Soc Mass Spectrom. 2024 Oct 5;35(12):2910–7. doi: 10.1021/jasms.4c00255 (PMC11622222; doi:10.1021/jasms.4c00255)
Supplement: Supplementary file 1 — js4c00255_si_001.pdf [file js4c00255_si_001.pdf]

Supplementary Materials for "Combined quantification and characterization of dissolved organic matter by liquid chromatography – mass spectrometry using charged aerosol detection".

Stacey L. Felgate[1], Elizabeth Jakobsson[2], Andrea Balderrama Subieta[1], Lars J. Tranvik[2], and Jeffrey A. Hawkes[1*]

[1]*Department of Chemistry, Uppsala University, Uppsala 751 23, Sweden*

[2]*Department of Ecology and Evolution, Uppsala University, Uppsala 752 36, Sweden*

[*]Corresponding author: Jeffrey A. Hawkes (Jeffrey.hawkes@kemi.uu.se)

<u>Contents</u>

Text S1. Description of sampling sites.
Text S2. Sample collection protocol.
Text S3. List of reagents.
Text S4. Solid phase extraction
Text S5. Quantification of DOC and SPE-DOC

Table S1. Locations of sampling sites.
Table S2. Peak assignment metrics for SRNOM injections.
Table S3. Details of the gradient used during reversed phase chromatographic separation.
Table S4. Water chemistry data (DOC, SPE-DOC, Extraction Efficiency, SPE-DOM, and % SPE-DOC.
Table S5. High resolution mass spectrometry peak metrics.
Table S6. Bray-Curtis dissimilarity among replicates within and between sampling occasions.

Figure S1. Assessment of sample carryover to blank
Figure S2. Calibration curve between injected SRNOM concentration and CAD peak area.
Figure S3: Formula assignment mass error summary
Figure S4: Chromatograms showing assignment coverage
Figure S5. Intensity of two internal standards for each sample, showing some ionization suppression in winter samples.
Figures S6 – S8. Plots of abundance, absorption and total assigned current at each site, plotted by season.

Text S1. Locations (latitude and longitude) and short descriptions for Sites 1 - 8.

The first site (Site 1) is small reed pond located on the outskirts of Uppsala. Lake Långsjön (Site 2) drains a mix of forested, agricultural, and urban land, and has a residence time of between 2 and 4 years. Lake Erken (Site 3) drains mostly forested land, and has a residence time of 7 years [1]. Elkoln (Site 4) is the northernmost gulf of Lake Mälaren, the third largest Lake in Sweden [2], and is fed by the River Fyris (Site 5) after it has flowed through Uppsala City Centre (Site 6). A smaller river draining agricultural fields (Site 7) and a drainage pond fed by a wetland (Site 8) were also included. Location details are provided in Table S1.

Text S2. Sample collection protocol

At each site, two pre-combusted (4 hrs at 450°C) 250 ml glass Duran® bottles were triple rinsed with sample water and filled with surface water directly from the waterbody. On return to the laboratory, each sample was vacuum filtered using a Nalgene® filter housing and a pre-combusted 47 mm diameter GF/F grade Whatman® glass fibre filter. A new filter was used for each sample, with the first 25 ml filtrate discarded. Samples for SPE and DOC quantification were then collected into pre-combusted 100 ml glass Duran® bottles and pre-combusted 40 ml glass vials fitted with PTFE liner caps, respectively, and stored at 4°C pending further analysis. SPE occurred within 24 hrs. DOC analysis occurred within 1 week.

Text S3. List of reagents

LC-MS grade methanol (Supelco LiChroSolv hypergrade for LC-MS), hydrochloric acid (37% HCl), and formic acid (FA) were obtained from VWR (AmalaR Normapur, VWR Sweden). Acetonitrile (ACN) was obtained from Sigma Aldrich (Supelco LiChroSolv hypergrade for LC-MS). Suwannee River natural organic matter (SRNOM) was obtained from the International Humic Substances Society (IHSS, Saint-Paul, USA; batch number 2R101N). Hippuric and fuscidic acid were obtained from Sigma Aldrich (Merck Life Science AB, Sweden).

Text S4. Solid phase extraction

100 mg Agilent PPL (styrene-divinylbenzene) cartridges [3] were pre-conditioned by flushing with 3 ml MeOH, soaking in MeOH overnight, then flushing with 3 ml acidified Milli-Q™ (0.1 % FA). Samples were acidified to a pH of 2 using 6M HCl then loaded into 60 ml syringes and left to gravity feed the cartridges. A second 3 ml acidified Milli-Q™ (0.1 % FA) flush was used to remove any salts before eluting the cartridges with 2 ml MeOH, approximately 1.8 ml of which was recovered into pre-combusted 2 ml amber vials. Sample vials were weighed on a balance before and after sample extraction, with the mass decrease (initial mass – end mass) multiplied by the density of water (0.997 mg mL^{-1} at 25 °C) to determine the extraction volume. Similarly, the eluted volume was determined by multiplying the mass increase (end mass – initial mass) by the density of MeOH (0.792 mg mL^{-1} at 25 °C). Eluate was stored upright at -20°C until analysis ~1 week later.

Text S5. Quantification of DOC and SPE-DOC

[DOC] and [SPE-DOC] were both analysed on a Shimadzu TOC-V analyser and quantified as non-purgeable organic carbon (NPOC) against an Ethylenediaminetetraacetic acid (EDTA) standard and Milli-Q™ blanks. Measurements were made in triplicate, and are reported as a mean ± standard deviation. All replicate measurements had a coefficient of variation of ≤ 2%, and the EDTA standard was within 5 % of the working solution.

Table S1. Locations of each sampling site

Site	Latitude (N)	Longitude (E)	Description	DOC concentration (winter)
Site 1	59°50'44.61"	17°35'58.42"	Reed pond	5.11
Site 2	60°02'31.97"	17°33'36.40"	Långsjön (lake)	6.17
Site 3	59°50'07.52"	18°37'57.89"	Erken (lake)	9.77
Site 4	59°46'56.35"	17°37'33.93"	Ekoln (lake)	10.84
Site 5	59°47'14.08"	17°39'44.05"	Fyrisån (river, mouth)	17.95
Site 6	59°51'15.91"	17°38'30.68"	Fyrisån (river, city)	17.14
Site 7	59°52'27.37"	17°23'52.59"	Agricultural river	22.02
Site 8	59°55'40.57"	17°20'48.95"	Wetland drainage pond	26.74

Table S2. Assignment metrics for SRNOM with differing injection amounts. At low injections, fewer peaks are assigned, and the average metrics (O/C, H/C and m/z) are quite different compared with higher injection amounts.

Injection amount (µg SRNOM)	number of peaks	sum intensity	O/C	H/C	m/z
1	2840	4.97	0.44	1.45	238.23
5	4105	6.39	0.54	1.20	334.91
10	4650	8.14	0.57	1.11	371.20
20	5030	10.52	0.58	1.07	392.09
30	5234	12.29	0.58	1.05	397.97
40	5373	13.60	0.58	1.05	401.09

Table S3. Details of the gradient used during reversed phase chromatographic separation.

Time Period (min)	Mobile Phase A (%)	Mobile Phase B (%)
0.0 – 1.0	100	0
1.0 – 11.0	100 -> 0	0 -> 100
11.0 – 11.5	0	100
11.5 – 12.0	100 -> 0	0 -> 100
12.0 – 15.0	100	0

Table S4. Water chemistry data, showing mean DOC, SPE-DOC, Extraction Efficiency, SPE-DOM, and SPE-DOC as a % of SPE-DOM (n = 2).

		**[DOC] (mg L^{-1})**	**[SPE-DOC] (mg L^{-1})**	**Efficiency (%)**	**[SPE-DOM] (mg L^{-1})**	**SPE-DOC %**
Site 1	Summer	6.17	3.55	57	8.34	43
	Winter	5.11	2.25	49	7.21	35
	Difference	*-1.06*	*-1.03*	*-8*	*-1.12*	*-8*
Site 2	Summer	5.98	2.98	50	7.51	40
	Winter	6.17	3.18	52	8.79	36
	Difference	*0.19*	*0.20*	*2*	*1.29*	*-4*
Site 3	Summer	9.86	5.78	59	13.82	42
	Winter	9.77	5.48	56	13.46	41
	Difference	*-0.09*	*-0.30*	*-3*	*-0.36*	*-1*
Site 4	Summer	11.89	5.80	49	19.09	31
	Winter	10.84	6.18	57	16.01	39
	Difference	*-1.05*	*0.39*	*8*	*-3.08*	*+8*
Site 5	Summer	11.94	5.99	50	16.36	37
	Winter	17.95	9.13	51	24.45	38
	Difference	*6.02*	*3.14*	*1*	*8.09*	*+1*
Site 6	Summer	12.63	7.27	58	18.68	39
	Winter	17.14	9.65	57	24.41	40
	Difference	*4.51*	*2.39*	*-1*	*5.73*	*+1*
Site 7	Summer	5.94	3.72	63	10.58	35
	Winter	22.02	12.14	55	32.09	38
	Difference	*16.08*	*8.42*	*-8*	*21.50*	*+3*
Site 8	Summer	18.12	11.00	61	26.66	41
	Winter	26.74	16.25	61	38.85	42
	Difference	*8.62*	*5.25*	*0*	*12.19*	*+1*

Table S5. High resolution mass spectrometry peak metrics showing mean number of peaks (Peaks) and the intensity weighted average of oxygen to carbon (O/C_{wa}), hydrogen to carbon (H/C_{wa}) and mass to charge (m/z_{wa}) ratios (n = 2).

		**Peaks**	**O/C_{wa}**	**H/C_{wa}**	**m/z_{wa}**
	Summer	5862	0.530	1.075	380.27
Site 1	Winter	5701	0.539	1.090	400.35
	Difference	*-161*	*0.009*	*0.015*	*20.08*
	Summer	5372	0.562	1.129	384.27
Site 2	Winter	5295	0.593	0.989	385.51
	Difference	*-77*	*0.031*	*-0.140*	*1.24*
	Summer	5630	0.582	1.078	398.17
Site 3	Winter	5539	0.578	1.076	402.90
	Difference	*-91*	*-0.004*	*-0.001*	*4.73*
	Summer	5519	0.573	1.032	410.59
Site 4	Winter	5546	0.577	1.022	422.07
	Difference	*27*	*0.004*	*-0.010*	*11.48*
	Summer	5860	0.573	1.032	405.76
Site 5	Winter	5124	0.575	1.022	429.47
	Difference	*-736*	*0.002*	*-0.010*	*23.71*
	Summer	5635	0.576	1.032	410.18
Site 6	Winter	5270	0.580	1.020	429.83
	Difference	*-365*	*0.005*	*-0.012*	*19.65*
	Summer	5555	0.559	1.053	412.20
Site 7	Winter	5331	0.579	1.012	433.14
	Difference	*-224*	*0.020*	*-0.041*	*20.94*
	Summer	5473	0.571	1.021	431.65
Site 8	Winter	5165	0.585	1.005	439.89
	Difference	*-308*	*0.014*	*-0.016*	*8.24*

Table S6. *Bray-Curtis dissimilarity among replicates within and between sampling occasions. For summer-winter dissimilarity values are means ± standard deviation across all four pairwise comparisons.*

	**Summer inter-dissimilarity (%)**	**Winter inter-dissimilarity (%)**	**Summer-winter dissimilarity (%)**
**# Comparisons**	1	1	4
Site 1	7.6	8.2	20.3 ± 0.8
Site 2	7.1	5.2	35.3 ± 0.2
Site 3	3.2	3.6	4.8 ± 0.9
Site 4	3.1	3.4	7.3 ± 0.4
Site 5	3.1	7.7	15.1 ± 0.9
Site 6	5.8	3.1	11.8 ± 0.7
Site 7	3.3	3.5	12.5 ± 0.2
Site 8	5.6	6.7	9.4 ± 1.5

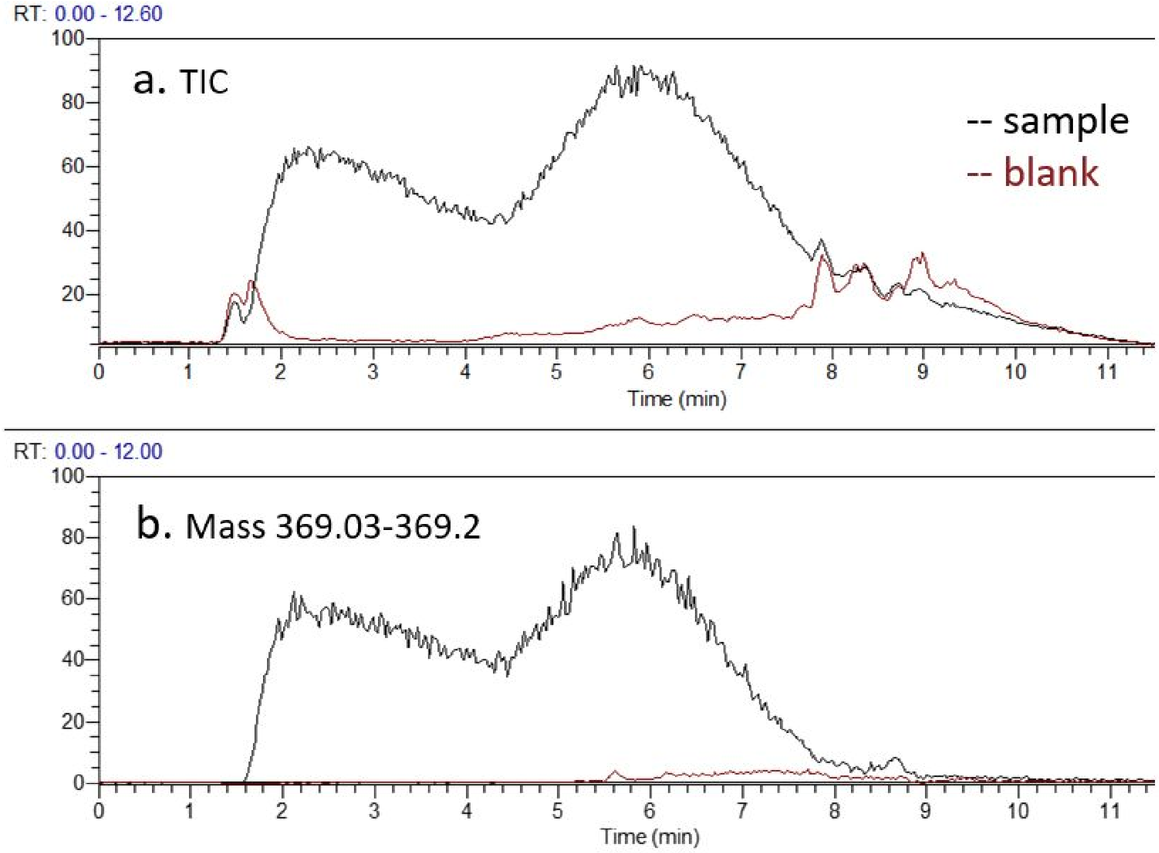

Figure S1: Plot of (a) TIC and (b) mass range 369.03-369.2, which contains several prominent peaks in DOM, in a sample (black) and a blank (red) that was analysed directly afterwards, showing minimal carryover of material.

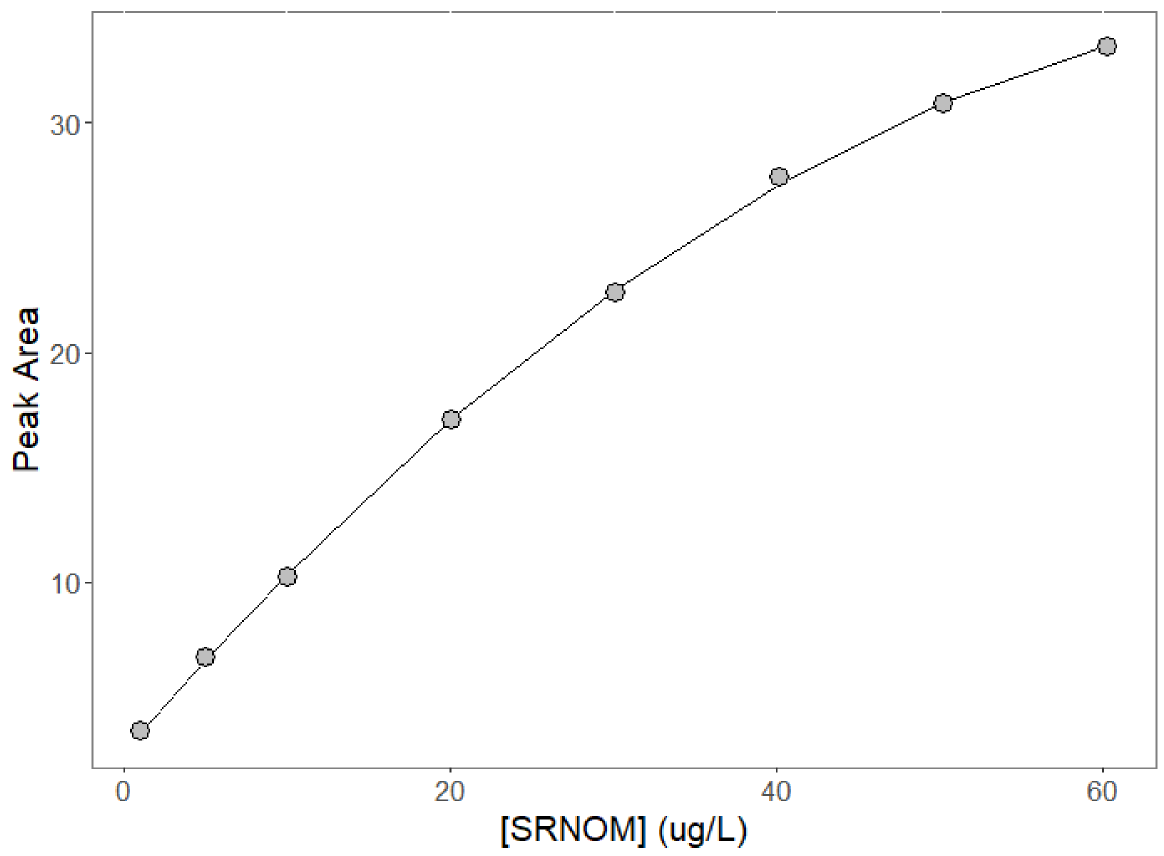

Figure S2. Calibration curve between injected SRNOM concentration and CAD peak area integrated between 1.5 and 11.5 mins.

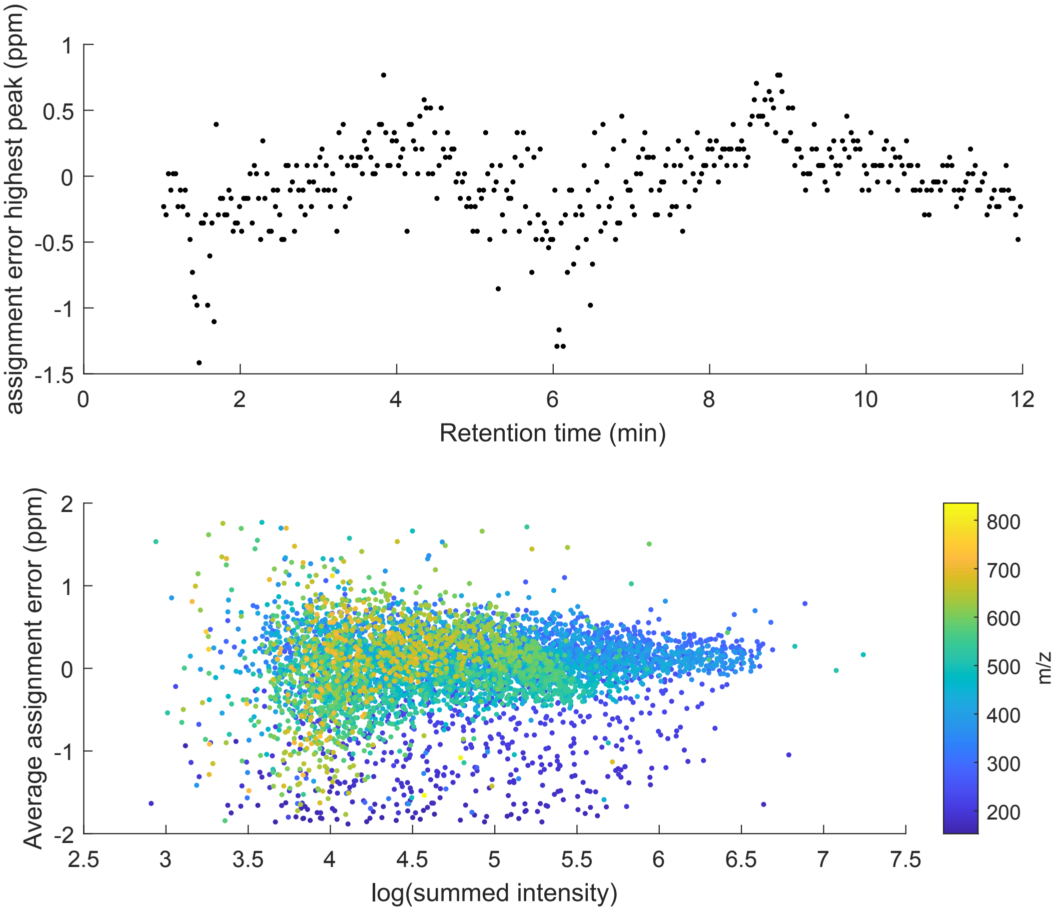

Figure S3: Formula assignment mass error summary for sample “CAD11_10A-20s_30ul_May2nd.mzXML” (replicate 1 of site 1, summer). The top plot shows the error over the chromatogram for the highest peak in this sample, and the lower plot shows the error (averaged over retention time) for all peaks, plotted vs. log(intensity) and with mass as color.

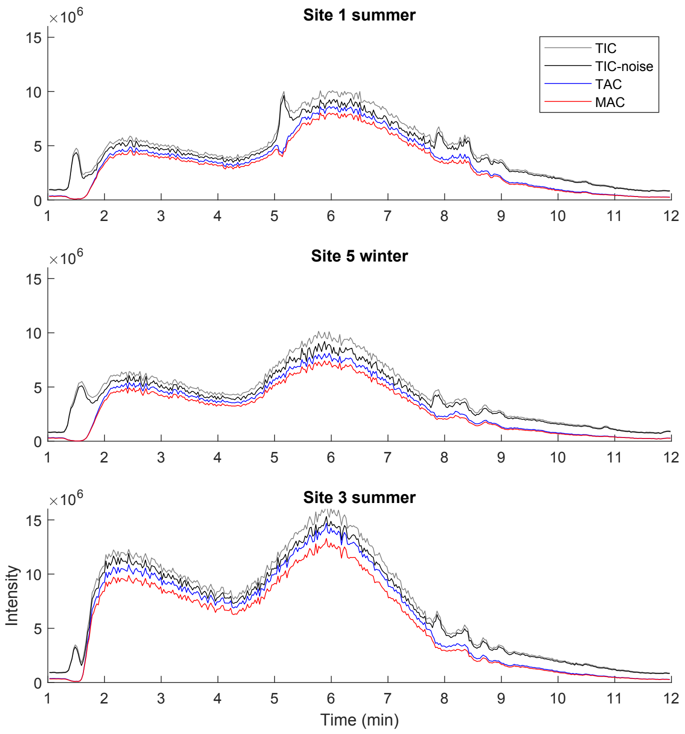

Figure S4: Chromatograms for three samples showing total ion current (grey), total ion current without noise (black), total assigned current (blue) and total assigned current without isotopologues (monoisotopic assigned current; MAC, red). Assignment coverage can be assessed as blue vs. black, and shows that most intensity was assigned a formula, even with the atom constraints used.

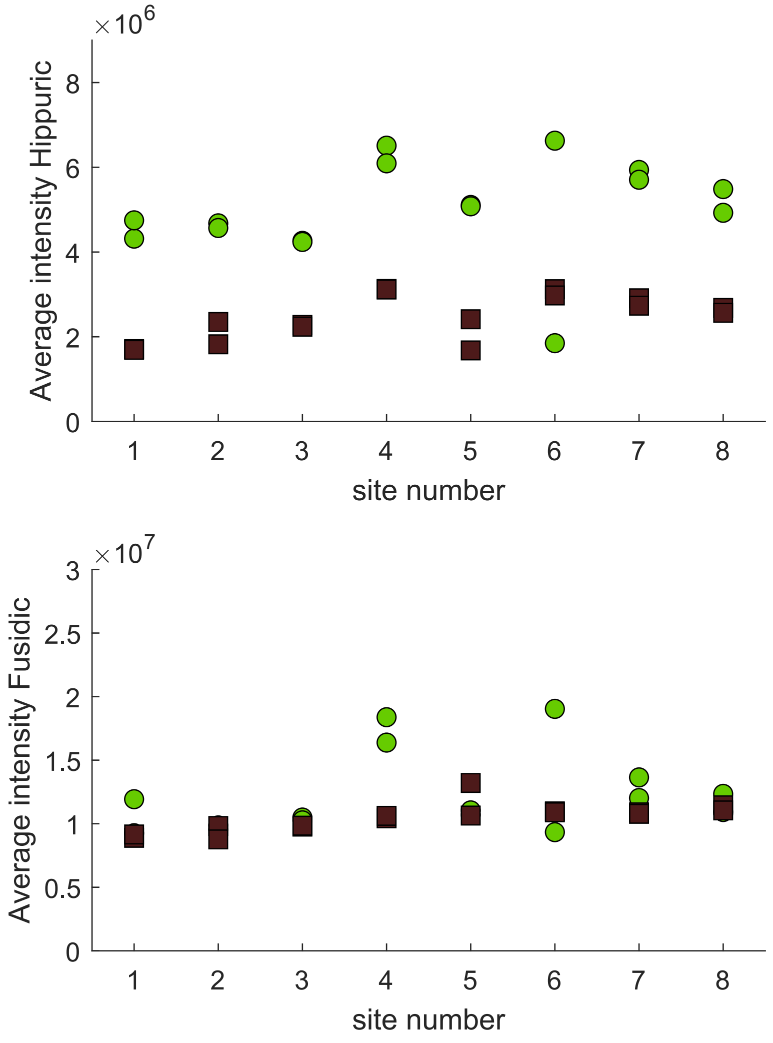

Figure S5: Obtained average LC-MS intensity for two internal standards, which are dissolved in the second (post column) pump's mobile phases, and should be present at constant and identical concentration across samples.

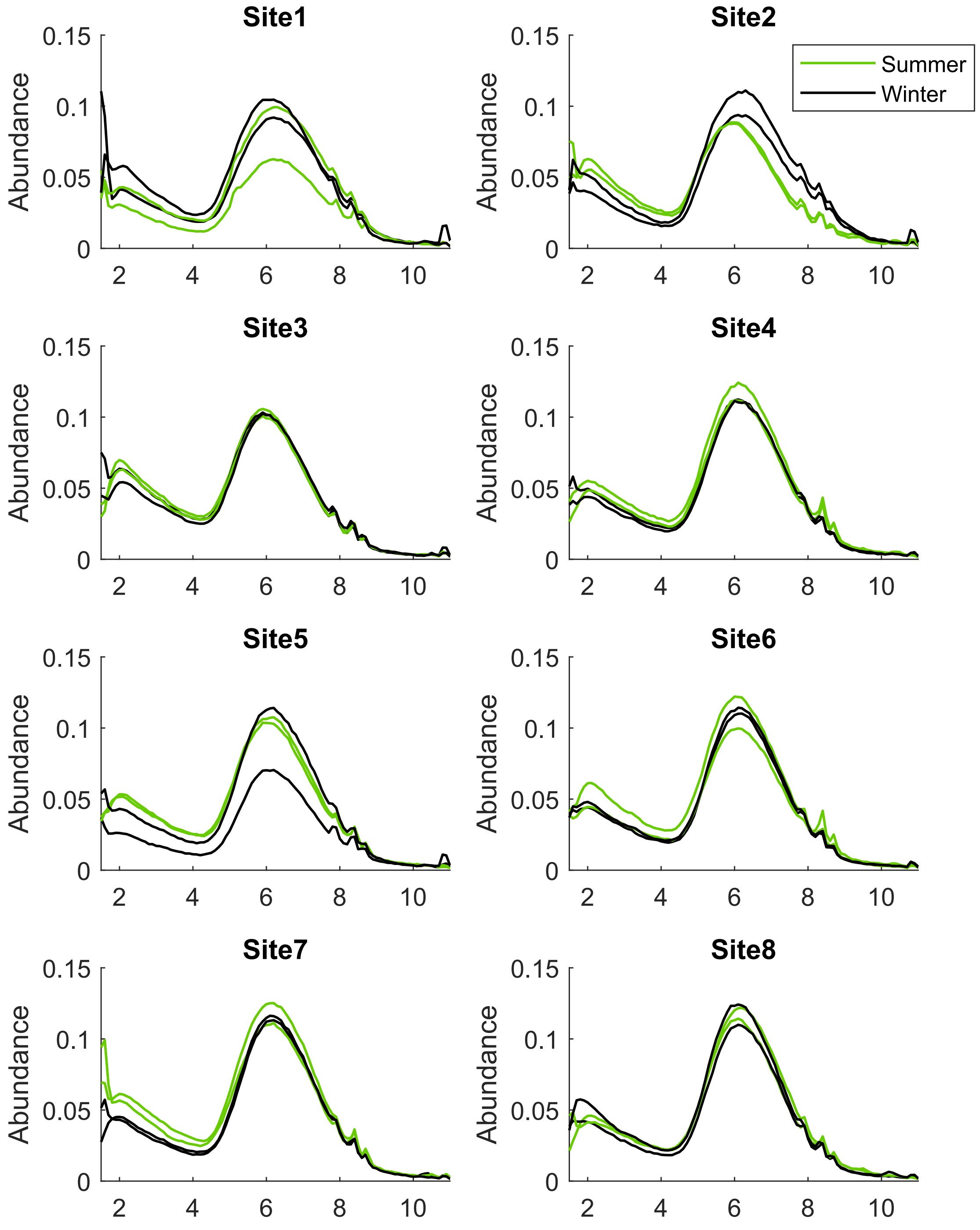

Figure S6: Chromatograms of charged aerosol detector signal (CAD, or abundance) for each site, plotted in colour by season and for each of two replicates.

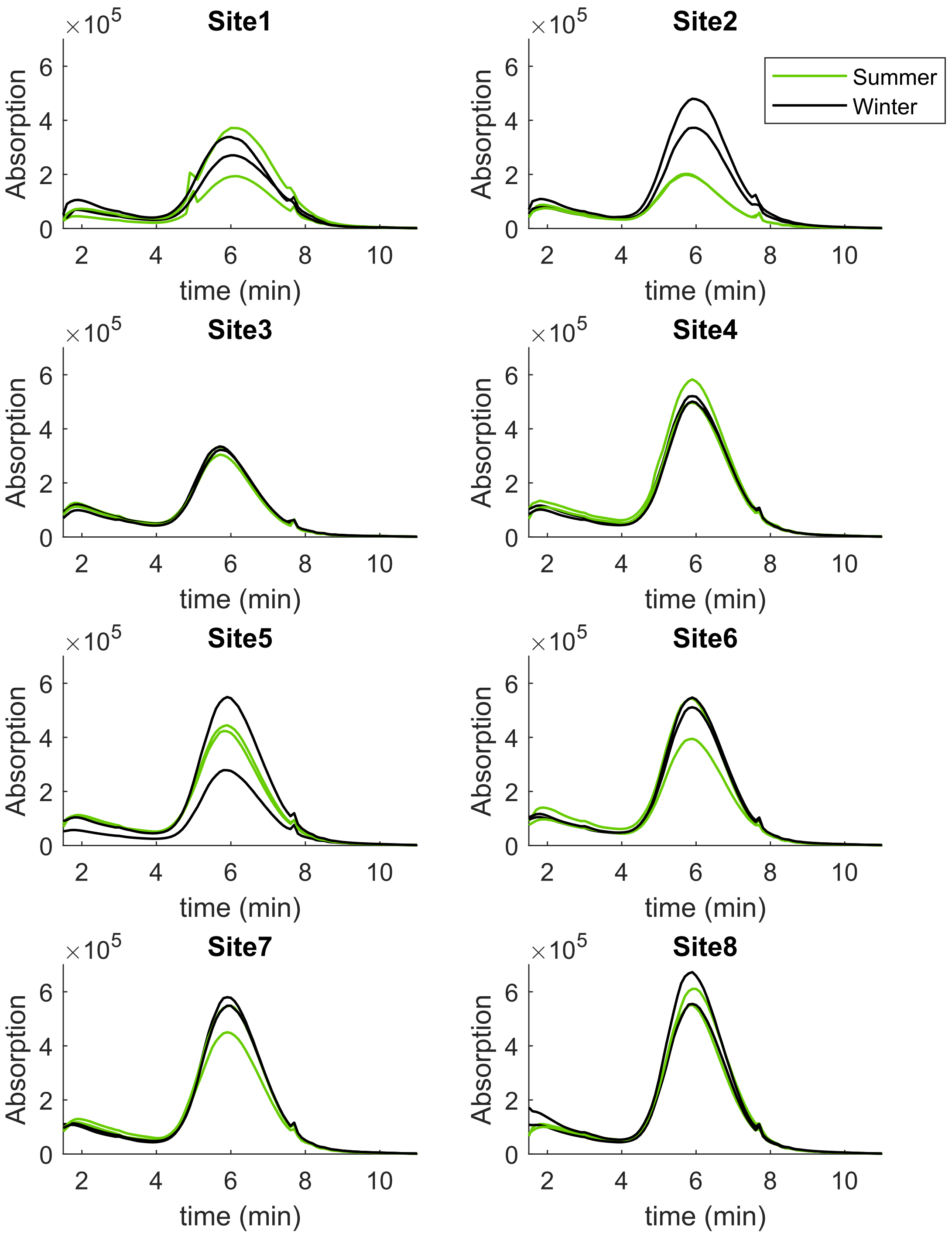

Figure S7: Chromatograms of UV absorption from the diode array detector (DAD) for each site, plotted in colour by season and for each of two replicates.

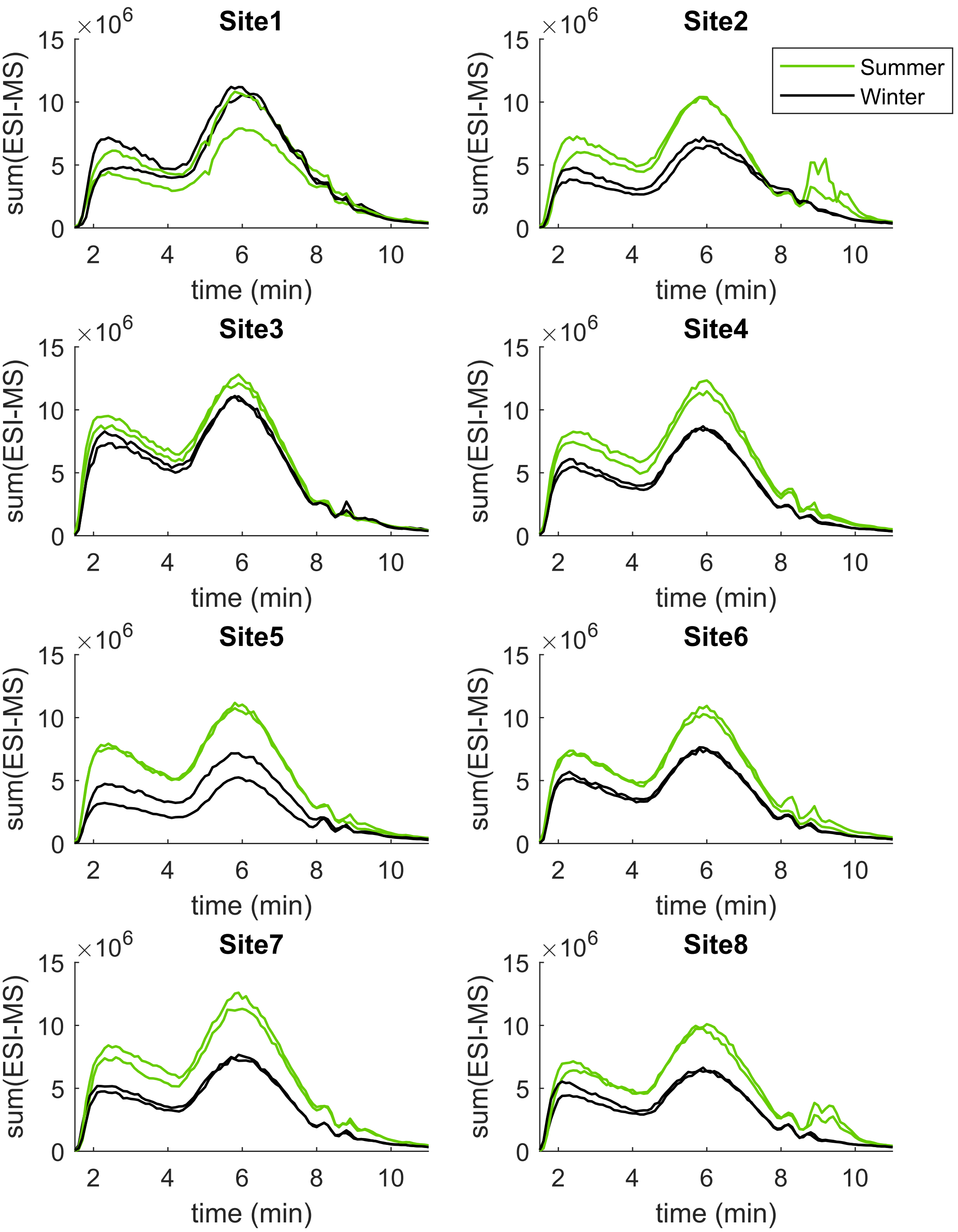

Figure S8: Chromatograms of total assigned current (TAC, or sum(ESI-MS)) for each site, plotted in colour by season and for each of two replicates.

References

(1) Malmaeus, J. M.; Blenckner, T.; Markensten, H.; Persson, I. Lake Phosphorus Dynamics and Climate Warming: A Mechanistic Model Approach. *Ecological Modelling* **2006**, *190* (1), 1–14. https://doi.org/10.1016/j.ecolmodel.2005.03.017.
(2) Weyhenmeyer, G. A.; Willén, E.; Sonesten, L. Effects of an Extreme Precipitation Event on Water Chemistry and Phytoplankton in the Swedish Lake Mälaren. **2004**, *9*, 12.
(3) Dittmar, T.; Koch, B.; Hertkorn, N.; Kattner, G. A Simple and Efficient Method for the Solid-Phase Extraction of Dissolved Organic Matter (SPE-DOM) from Seawater. *Limnology and Oceanography: Methods* **2008**, *6* (6), 230–235. https://doi.org/10.4319/lom.2008.6.230.
